# Comparing the Effects of a Pine (*Pinus* *radiata* D. Don) Bark Extract with a Quebracho (*Schinopsis* *balansae* Engl.) Extract on Methane Production and In Vitro Rumen Fermentation Parameters

**DOI:** 10.3390/ani12091080

**Published:** 2022-04-21

**Authors:** Nelson Vera, Constanza Gutiérrez-Gómez, Pamela Williams, Rodrigo Allende, Cecilia Fuentealba, Jorge Ávila-Stagno

**Affiliations:** 1 Departamento de Ciencia Animal, Facultad de Ciencias Veterinarias, Universidad de Concepción, Campus Chillán, Chillán 3812120, Chile; nevera@udec.cl (N.V.); constgutierrez@udec.cl (C.G.-G.); rallende@udec.cl (R.A.); 2 Departamento de Producción Animal, Facultad de Agronomía, Universidad de Concepción, Campus Chillán, Chillán 3812120, Chile; pamelawilliams@udec.cl; 3 Unidad de Desarrollo Tecnológico, Universidad de Concepción, Coronel 4190000, Chile; c.fuentealba@udt.cl; 4 Centro Nacional de Excelencia para la Industria de la Madera (CENAMAD), Pontificia Universidad Católica de Chile, Santiago 7820436, Chile

**Keywords:** greenhouse gases, mitigation, tannin, livestock, polyphenol, ruminant

## Abstract

**Simple Summary:**

Livestock production systems are responsible for 37 and 64% of total anthropogenic methane (CH_4_) and ammonia (NH_3_) overall planet emissions, respectively. Due to the growing demand for meat and milk, mitigating their environmental impact is a major concern. A novel tannin-rich phenolic extract from radiata pine bark (PBE) has the potential to reduce CH_4_ and NH_3_ nitrogen (NH_3_-N) production and modulate rumen fermentation but has not been compared with other commercial phenolic extracts. This study compared PBE with a quebracho extract (QTE). Both extracts decreased butyrate proportion, CH_4_, total volatile fatty acids, NH_3_-N production, and increased acetate proportion. Inclusion of QTE increased the propionate proportion but decreased DM disappearance. Results indicate that PBE has the potential to contribute to sustainable livestock production; however, further in vivo studies are needed to verify our results.

**Abstract:**

The aim of this study was to compare the effects of a pine (*Pinus radiata* D. Don) bark extract (PBE) with a quebracho (*Schinopsis balansae* Engl.) extract (QTE) on methane (CH_4_) production and in vitro rumen fermentation parameters. A forage diet supplemented with PBE or QTE (0, 2 and 4% dry matter (DM) basis) was incubated for 24 h to determine in vitro DM disappearance (IVDMD), CH_4_, volatile fatty acids (VFA), and ammonia nitrogen (NH_3_-N) production. Differences were analyzed using Tukey’s test, orthogonal contrasts, hierarchical clustering heatmap (HCH), and principal component analysis (PCA). Both extracts (4% DM) decreased butyrate (Bu; *p* = 0.001), CH_4_ (*p* = 0.005), total VFA (*p* < 0.001), and NH_3_-N (*p* = 0.006) production and increased acetate (Ac; *p* = 0.003) without affecting the partitioning factor (*p* = 0.095). Propionate (Pr; *p* = 0.016) was increased, whereas IVDMD (*p* = 0.041) was decreased with QTE (4% DM). The inclusion of QTE (2% DM) decreased CH_4_ production (*p* *=* 0.005) and the (Ac + Bu)/Pr ratio (*p* *=* 0.003), whereas PBE (2% DM) decreased the NH_3_-N (*p* *=* 0.006) and total VFA production (*p* < 0.001). The HCH and PCA indicate a negative correlation (r = −0.93; *p* < 0.001) between CH_4_ production and tannins. In conclusion, PBE shares many of the effects generated by QTE on ruminal fermentation, although the magnitude of these effects depends on concentration. The PBE could be used as an additive in ruminant diets to reduce CH_4_ and NH_3_-N production without reducing IVDMD or increasing propionate, but further in vivo studies are required to clarify its effects on animal production.

## 1. Introduction

Livestock systems are responsible for 37, 65 and 64% of total anthropogenic methane (CH_4_), nitrous oxide (N_2_O) and ammonia (NH_3_) overall planet emissions, respectively [1]. Due to the growing demand and production of meat and milk, driven by the growing world population, the environmental impact of ruminant livestock has increased [2]. Carbon dioxide (CO_2_), CH_4_ and N_2_O are greenhouse gases (GHGs) that have increased their atmospheric concentrations during the last century and relate ruminants to global warming [3,4]. Hence, the exploration of local strategies that improve the efficiency of ruminant production and mitigate GHG emissions is urgently needed [5].

Enteric fermentation generates CH_4_, the main contribution of ruminants to GHG [6], which represents an energy waste [5]. Additionally, different nitrogen (N) fractions are lost by protein degradation in the rumen as up to 50% is degraded to NH_3_, which is excreted in urine and can be oxidized to N_2_O [7]. Rumen fermentation can be manipulated to improve dietary energy and protein efficiency, mitigate CH_4_ emissions and reduce NH_3_ excretion into the environment [4,5]. Among strategies to manipulate ruminal fermentation, only those that have a positive economic impact on production will be implemented [8]. The use of growth promoters, antimicrobials and hormones in ruminant diets has been controversial and is prohibited in some markets, such as the European Union, due to the potential development of antibiotic-resistant microorganisms or the presence of chemical residues in final products [7,9]. Consumers’ negative perception of chemical additives has stimulated research on plants, extracts or secondary metabolites (e.g., polyphenols) to modify ruminal fermentation, improve productive performance [7,10] and reduce livestock environmental impact by decreasing CH_4_ production and N excretion in urine [3,11].

Tannins are polyphenolic compounds and an important group of secondary plant metabolites with a variety of molecular weights and structures [12] that in the past were considered antinutritional factors due to their aversive and toxic effects on herbivores [13]. Diets containing high amounts of tannins (>50 g kg^−1^ dry matter (DM)) [14] can reduce voluntary DM intake and animal production. However, it is now recognized that some tannins in low (<30 g kg^−1^ DM) or moderate (30–50 g kg^−1^ DM) concentrations can have positive effects on the environment, cattle nutrition and production [11,15]. Tannins can bind proteins, structural carbohydrates and starches and affect the rumen microbiome [9,16]. As such, they could have a positive effect on ruminant health by bloat prevention, endoparasite control, and improved immune responses and animal performance [10,13]. In addition, tannins can increase the conjugated linoleic acid concentrations in meat and milk [12] by inhibiting the last step of the biohydrogenation process of dietary unsaturated fatty acids [17] or by promoting its initial steps [18]. Furthermore, tannins can reduce the environmental impact of ruminants by decreasing dietary protein degradation and reducing their CH_4_ enteric production [10].

An extract from quebracho (QTE; *Schinopsis balansae* and *S. lorentzii*), an endangered tree from the Argentinian Chaco, is still commercially available and possibly the most studied source of tannins [5,12,14]. However, the use of quebracho is facing reduced availability and increased costs given its conservation condition. Alternatively, a polyphenolic extract from *Pinus radiata* bark (PBE), a very abundant by-product from the forest industry in Chile, contains a mixture of flavonoids, stilbenoids and condensed tannins [19]. This extract has been scarcely studied but has shown to reduce the NH_3_ nitrogen (NH_3_-N) concentration without affecting diet digestibility [20]. However, PBE has not been compared to other commercial phenolic extracts, such as QTE, a commonly used source of polyphenols in Latin America, nor has it been tested in forage diets. As such, the use of PBE in a ruminant diet based on forage will expand the understanding of the effects of this polyphenolic extract. Therefore, our hypothesis was that the inclusion of PBE as a feed additive in forage diets can reduce CH_4_ production and modulate ruminal fermentation parameters without affecting diet digestibility as compared with the inclusion of QTE, and that the effects depend on PBE or QTE concentration in the diets. The objective of this study aimed to compare in forage diets (forage-to-concentrate ratio of 60/40) the inclusion of PBE and QTE in concentrations of 0, 2 and 4% DM on CH_4_ production, ruminal fermentation parameters (pH, NH_3_-N and total volatile fatty acids (VFA)) and in vitro DM disappearance (IVDMD) using the in vitro gas production technique.

## 2. Materials and Methods

### 2.1. Study Location

This assay was performed at the Universidad de Concepción (UdeC), Livestock Systems and Nutrition Laboratory, Chillán, Chile. The care, management and assistance of the cows during the course of the study were certified by the ethics and animal welfare committee, UdeC (CBE 28–2019).

### 2.2. Plant Extracts and Experimental Substrates

This in vitro study used two plant tannin extracts at feasible concentrations for in vivo conditions. Therefore, the concentration could not be >5% DM, since tannins can decrease feed intake [11,14]. Concentrations were based on previous studies where the addition of tannin modulates ruminal fermentation without negative effects [11,14,20,21]. The experimental substrates are described in Table 1. The substrates were high in crude protein (CP) to simulate the utilization of forages with high contents of rapidly degradable protein [3] in spring supplemented grazing systems in Chile. The substrates for incubation were a mixed ryegrass (*Lolium perenne*) and clover (*Trifolium repens*) hay, soybean meal and corn grain in a 60:15:25 ratio, which were progressively replaced with the plant extracts. All ingredients were finely ground to 2 mm using a grain mill (Breuer, Temuco, Chile) before including PBE or QTE at concentrations of 0, 2 and 4% DM and thereafter blended. The PBE was produced at the Technological Development Unit of the UdeC by the methanolic extraction of radiata pine (*P. radiata*) bark. This extract contains 38.0% DM with a total tannin (TT) concentration ≥4.35% [19], which is not commercially available so far due to the fact that it is in the early stages of development and its impact on animal performance is unknown. In contrast, the QTE (*S. balansae*; MGM, Unitan, Buenos Aires, Argentina) was a commercial powder containing ≥7.96% TT.

### 2.3. Animals, Rumen Inoculum and Incubation

Rumen fluid was obtained and combined from two adult non-lactating rumen-cannulated Aberdeen Angus cows (500-kg body weight) that were fed on an ad libitum basis with a diet composed of mixed hay (*L. perenne* with *T. repens*), ground corn, soybean meal and a vitamin-mineral supplement in a 60:15:10:5 ratio, respectively, formulated to be adequate in all nutritional requirements of an adult cow weighing 500 kg according to NASEM [22]. Cows were fed daily at 7 a.m. and 5 p.m. with permanent access to water.

Rumen fluid (500 mL) was collected 2 h after the morning feeding, filtered through four layers of cheesecloth into a preheated thermal flask at 39 °C and immediately carried to the laboratory. The inoculum was prepared by mixing the rumen fluid with a mineral buffer [23] in a 1/3 ratio (*v*/*v*). All reagents were analytical grade, and solutions were prepared with demineralized water.

A 500 mg sample of each substrate was weighed into a filter bag (F57; Ankom Technology Corp., Macedon, NY, USA), and each substrate bag was placed individually in a 50 mL amber glass bottle, fitted with a rubber stopper [8]. Three repetitions were incubated per treatment (*n* = 5) and sampling time (6, 12 and 24 h). Two blanks (no substrate) were included per sampling time to calculate net gas production (GP) and the IVDMD.

Bottles were preheated (39 °C), filled with inoculum (25 mL) and gassed with CO_2_ before sealing with rubber stoppers. Thereafter, bottles were placed in an orbital shaker (Heidolph Unimax, Schwabach, Germany) set to 90 oscillations/min, and incubated at 39 °C for 24 h (Forma TM Series II 3110 Water–Jacketed CO_2_ Incubator, Thermo Fisher Scientific, Waltham, MA, USA) [8]. The incubations (runs) were repeated 3 times during different weeks, resulting in a total bottle number of 153 ((5 treatments × 3 replicates + 2 blanks bottles) × 3 sampling times × 3 runs).

### 2.4. IVDMD, Gas and CH_4_ Production Measurements

Gas samples (15 mL) were collected at 6, 12 and 24 h of incubation from each bottle and immediately injected into a vacuumed flask (5.9 mL; Labco Exetainer, Lampeter, UK). The GP from each bottle was then measured by water displacement [24] in a graduated pipette (25 mL). Filter bags were removed, washed with distilled water and dried at 60 °C for 24 h to estimate IVDMD [8].

### 2.5. Determination of Culture pH, NH_3_-N and VFA

The pH of the inoculum of each bottle was measured using a digital pH meter (Thermo Scientific Orion Star A121, Waltham, MA, USA), and incubation fluid was sampled (2 mL) in a vial with a screw cap (Biologix Res. Co., Lenexa, KS, USA) with 150 μL of trichloroacetic acid (0.65 *w*/*v*) to determine the NH_3_-N concentration. Another sample was collected in a vial with 300 μL of metaphosphoric acid (0.25 *w*/*v*) to determine the VFA concentrations. At the start of each incubation (0 h), inoculum samples were collected, analyzed, and used to calculate net NH_3_-N and the VFA concentration [8].

### 2.6. Substrate Analysis and Calculations

Substrates were analysed according to AOAC [25] methods for DM (#934.01), Ash (#942.05), CP (#954.01), ether extract (EE; #920.39) and acid detergent fiber (ADF; #973.18) at the Animal Nutrition Laboratory (UdeC). Neutral detergent fiber (NDF) was determined by Mertens [26]. For the PBE, DM and NDF were determined by the aforementioned procedures.

Organic matter (OM) was estimated as 100–Ash; the hemicellulose (HC) was estimated as NDF–ADF, and non-fibrous carbohydrates (NFC) were estimated as 100–CP–EE–NDF–Ash.

Gas samples were analysed for CH_4_ concentration using an Agilent 7890B Gas Chromatograph (GC) System (Agilent Technologies, Santa Clara, CA, USA), with helium (He) as a carrier gas at 1.33 mL min^−1^. The GC was equipped with a GS–CarbonPLOT 30-m column and thermal conductivity detector (Agilent Technologies, Milan, Italy). The column oven, injector and detector temperature were set to 35, 185 and 150 °C, respectively. A 2 mL gas subsample was taken from each exetainer vial and injected into the GC manually. The CH_4_ gas used to prepare the standards was of analytical quality (99.5%; Linde Group, Santiago, Chile). The stock CH_4_ gas was diluted with N gas (Linde Group, Santiago, Chile) at room temperature (≈22–24 °C) to obtain standards of 15, 10, 7.5, 5.0, 2.5 and 1.0% of CH_4_.

The NH_3_-N concentrations were read at 625 nm in a UV-VIS Spectrophotometer Pharo 300 Spectroquant (Merck, Darmstadt, Germany). To determine VFA concentrations, liquid samples from each screw cap vial were filtered through 0.45 µm pore–sized filters, and a 1 µL liquid subsample was taken and injected into the GC manually. The GC was an Agilent 7890B GC System (Agilent Technologies, Santa Clara, CA, USA) equipped with a flame ionization detector and a 30 m column (DB–FFAP, Agilent Technologies, Milan, Italy), using He as the gas carrier (flow rate 2.0 mL min^−1^). The GC oven was programmed with an initial temperature of 150 °C and increased at a 5 °C min^−1^ rate until it reached the final temperature of 195 °C. This temperature was held for 5 min, and the reaction continued for 8 min. The temperature of the injector and detector port were 225 °C and 250 °C, respectively. Concentrations were determined by comparing the retention time and peak area with a VFA Supelco standard by means of the ChemStation v. 3.2 software (Agilent Technologies, Santa Clara, CA, USA). The total VFA concentration was calculated as the sum of acetic, propionic and butyric acids concentrations in the ruminal fluid. The molar proportion of each VFA in relation to the total VFA concentration was also calculated.

The potentially degradable fraction (*B*; %), relative degradation rate (*C*; h^−1^), and the constant microbial efficiency factor (*A*) were calculated using the Lavrenčič et al. [27] model:

IVDMD = *B* exp(–*C* exp(–*At*))

The calculated parameters (*B*, *C* and *A*) were used to estimate the first and second derivatives of the Gompertz model to obtain the maximum degradation rate (MDR) and the time of maximum degradation rate (*T*_MDR_), allowing for a greater comparative analysis of PBE and QTE as feed supplement [27].

The asymptotic (*b*) gas (mL g^−1^ incubated DM) or CH_4_ (mg g^−1^ incubated DM) production, production rate (*c*) and *Lag* were estimated using the Schofield et al. [28] model:

Gas or CH_4_ production = *b* exp(−exp(1 − *c* (*t* − *Lag*)))

The parameters *b*, *c* and *Lag* were used to estimate the half-life (*t*_1/2_; h), time taken for gas or CH_4_ production to reach 50% of its *b* value as well as the average production rate (AR), which is the average *c* value between the incubation starting and the *t*_1/2_ [29].

To assess the efficiency of fermentation at 24 h of incubation, the partitioning factor (PF) value was determined as the ratio between degraded DM (mg) and net gas volume (mL) [30]. Gas and CH_4_ yields were calculated as the volume of net gas (mL) or CH_4_ (mg) at every sampling time, divided by its corresponding degraded DM (g).

### 2.7. Statistical Analyses

Values measured from the three bottles incubated for each run, sampling time, and treatment were averaged before statistical analysis and treated as the statistical unit. Data were analysed with the statistical software Stata 16 (Stata Corporation, College Station, TX, USA). The Shapiro–Wilk and Levene’s tests were applied to all data to analyze variance normality and homogeneity, and outliers were detected and removed. The statistical units were analysed by ANOVA using a randomized complete block design according to the following model:

Y*_ijk_* = µ + α*_i_* + δ*_j_* + β*_k_* + ε*_ijk_*

where Y*_ijk_* is every observation; μ is the general mean of observations; α*_i_* is the fixed effect of the extracts (*i* = control, PBE, or QTE); δ*_j_* is the fixed effect of concentration (*j* = 0, 2 and 4% DM); β*_k_* is the random effect of incubation run (*k* = 1, 2 and 3); and ε*_ijk_* is the error. Differences between treatment means were determined using Tukey’s test, with significance declared at *p* < 0.05. Additionally, the significance of orthogonal contrasts was calculated for control vs. plant extracts (effect of the extract supplementation), PBE vs. QTE (effect of the plant extract type), and concentration of plant extracts at 0% DM vs. 2% DM vs. 4% DM (effect of concentration of plant extracts). Pearson correlation coefficients and hierarchical clustering heatmap (HCH) analysis were used to establish the qualitative assessment of the substrates’ composition and ruminal fermentation parameters to investigate the similarities between the plant extracts. Principal component analysis (PCA) was conducted to analyze the spatial patterns of association between substrate composition and ruminal fermentation parameters. The Kaiser–Meyer–Olkin (KMO) measure of sampling adequacy and Bartlett’s test of sphericity (BTS) were applied to analyze the data adequacy to perform the PCA. The value of KMO ranges from 0 to 1, and the accepted index should be >0.6 if variables are sufficiently interdependent for the PCA to be useful, whereas the BTS should be significant with *p* < 0.05 [31]. The calculated KMO was 0.627, and the BTS was significant (*p* < 0.001).

## 3. Results

### 3.1. IVDMD, Gas and CH_4_ Production Kinetics

The DM potentially degradable fraction (%) was lower (*p* = 0.040) for QTE than PBE at 4% DM (Table 2), whereas the MDR (% h^−1^) was lower (*p* = 0.014) for QTE than PBE at 2 and 4% DM. However, the relative degradation rate (h^−1^) and the *T*_MDR_ (h) decreased (*p* = 0.004 and *p* = 0.017, respectively) with 4% of PBE as compared to the control (CTL) substrate, whereas the microbial efficiency constant was lower (*p* = 0.013) with PBE as compared to QTE. However, they did not differ from CTL at any concentration. On the other hand, the GP rate (h^−1^) decreased (*p* = 0.008) with a 4% DM concentration of PBE and QTE as compared to CTL; lag (h) of GP only decreased (*p* = 0.008) with QTE (4% DM), whereas the *t*_1/2_ (h) only increased (*p <* 0.001) with PBE (4% DM), but asymptotic GP (mL g^−1^ incubated DM) and the AR (mL g^−1^ incubated DM h^−1^) were unaffected (*p* = 0.512 and *p* = 0.331, respectively). Asymptotic CH_4_ production (mg g^−1^ incubated DM) decreased with 4% DM of QTE (*p* = 0.039), whereas the AR (mg g^−1^ incubated DM h^−1^) decreased (*p* = 0.014) with 4% DM of PBE. By contrast, the lag (h) and the *t*_1/2_ (h) were increased (*p* = 0.014 and *p* = 0.007, respectively) with PBE as compared to QTE at 4% DM. The rate of CH_4_ production (h^−1^) was unaffected (*p* = 0.496).

### 3.2. IVDMD, Gas and CH_4_ Outputs

At 6 h of incubation, the IVDMD (%) was lower (*p* = 0.023) with PBE at 4% DM compared to CTL (Table 3). However, at 12 h, it was reduced (*p* = 0.004) by both extracts at 4% DM, but at 24 h, it was reduced (*p* = 0.041) only with 4% DM of QTE as compared to CTL.

The net GP (mL) at 6 h was lower (*p* < 0.001) with PBE at 2 and 4% DM. At 12 h, it decreased (*p* = 0.003) with PBE (2 and 4% DM) and QTE (4% DM), but at 24 h, there were no differences (*p* = 0.130). The GP (mL g^−1^ incubated DM) response was similar to IVDMD, with a reduction at 6 h of incubation (*p* = 0.011) with PBE (4% DM) and at 12 h (*p* = 0.002) with PBE and QTE (4% DM). However, no differences (*p* = 0.743) were recorded at 24 h. The GY (mL g^−1^ degraded DM) was only decreased (*p* = 0.027) at 6 h with PBE (4% DM). There were no differences at 12 and 24 h between treatments (*p* = 0.279 and *p* = 0.369, respectively).

At 6 and 12 h of incubation, the net CH_4_ production (mg) was lower (*p <* 0.001) in PBE at 2 and 4% DM, whereas at 24 h, it decreased (*p <* 0.001) with PBE (2 and 4% DM) and QTE (4% DM). Furthermore, the ratio of mg CH_4_ mL^−1^ gas was reduced (*p* ≤ 0.038) with PBE (4% DM) at 6, 12 and 24 h. The CH_4_ production (mg g^−1^ incubated DM) was decreased by PBE (4% DM) at 6 and 12 h of incubation (*p* = 0.019 and *p* = 0.007, respectively), whereas at 24 h, it was lower (*p <* 0.001) with PBE (4% DM) and with QTE (2 and 4% DM). However, the in vitro CH_4_ yield (mg g^−1^ degraded DM) was reduced (*p* = 0.023) with PBE (4% DM) at 6 h but was unaffected (*p* ≥ 0.199) at 12 and 24 h.

### 3.3. In Vitro Ruminal Fermentation Parameters

The in vitro NH_3_-N production (mg dL^−1^) decreased (*p* = 0.006) with PBE (2 and 4% DM) and QTE (4% DM) at 24 h of incubation (Table 4). Incubation pH was lower (*p* = 0.031) for QTE as compared to PBE (4% DM), but did not differ from CTL, whereas the PF_24_ (mg degraded DM mL^−1^ gas) was unaffected (*p* = 0.095) by the extracts. Total VFA (m*M*) production decreased (*p* < 0.001) with PBE (2 and 4% DM) and QTE (4% DM), but in different magnitudes. Butyrate proportion (%) decreased (*p* = 0.001) with PBE (4% DM) and QTE (2 and 4% DM), acetate (%) increased (*p* = 0.003) with PBE and QTE at 4% DM, and propionate (%) increased (*p* = 0.016) only with 4% DM of QTE. The acetate-to-propionate (Ac/Pr) ratio was greater (*p* = 0.017) with 4% DM of PBE than with 2 and 4% DM of QTE, although they did not differ from CTL, whereas the sum of the acetate-and-butyrate-to-propionate ((Ac + Bu)/Pr) ratio was decreased (*p* = 0.003) with QTE (2 and 4% DM) compared to the CTL.

### 3.4. Pearson Correlation Coefficients and Hierarchical Clustering Analysis

**Figure 1:**
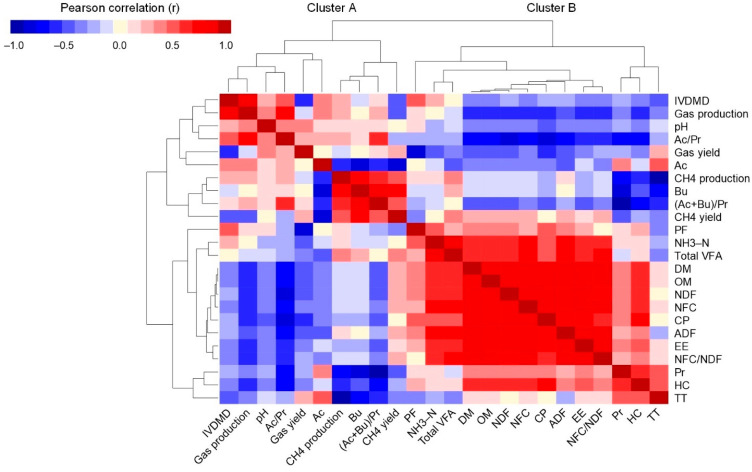
Hierarchical clustering heatmap of Pearson correlation coefficients between substrate composition and ruminal fermentation parameters after 24 h in vitro ruminal incubation. Darker red indicates a higher positive correlation (r → 1), a darker blue indicates a higher negative correlation (r → −1), and white indicates a lack of correlation (r ≅ 0). Dendrograms represent the hierarchical clustering of variables based on the correlations among them. (Ac + Bu)/Pr = sum of acetate-and-butyrate-to-propionate ratio; Ac/Pr = acetate-to-propionate ratio; Ac = acetate; ADF = acid detergent fiber; Bu = butyrate; CH_4_ = methane; CP = crude protein; DM = dry matter; EE = ether extract; HC = hemicellulose; IVDMD = in vitro dry matter disappearance; NDF = neutral detergent fiber; NFC = non–fibrous carbohydrates; NFC/NDF = ratio of non-fibrous carbohydrates to neutral detergent fibers; NH_3_-N = ammonia nitrogen; OM = organic matter; PBE = pine bark extract; PF = partitioning factor; Pr = propionate; QTE = quebracho extract; TT = total tannin; VFA = volatile fatty acid.

The HCH of Pearson correlation coefficients among the substrates’ composition and ruminal fermentation parameters after 24 h of incubation are shown in Figure 1. The TT content showed a high and negative correlation with CH_4_ production (r = −0.93; *p* < 0.001), a moderate negative correlation with butyrate (r = −0.67; *p* = 0.006) and (Ac + Bu)/Pr ratio (r = −0.61; *p* = 0.015), and a moderate positive correlation with propionate (r = 0.62; *p* = 0.014) and acetate (r = 0.52; *p* = 0.045). There was high positive correlation between NH_3_-N production and ADF (r = 0.81; *p* < 0.001), NFC (r = 0.76; *p* = 0.001), OM (r = 0.75; *p* = 0.001), DM (r = 0.74; *p* = 0.002), the ratio of non-fibrous carbohydrates to neutral detergent fibers (NFC/NDF) (r = 0.74; *p* = 0.002), NDF (r = 0.73; *p* = 0.002) and EE (r = 0.72; *p* = 0.003), but a moderate positive correlation with CP (r = 0.62; *p* = 0.013). A high positive correlation was observed between total VFA production and ADF (r = 0.85; *p* < 0.001), NFC/NDF (r = 0.78; *p* = 0.001), NFC (r = 0.77; *p* = 0.001), OM (r = 0.75; *p* = 0.001), DM (r = 0.74; *p* = 0.002) and NDF (r = 0.74; *p* = 0.002), but there was only a moderate positive correlation with EE (r = 0.68; *p* = 0.005) and CP (r = 0.57; *p* = 0.026). Furthermore, the Ac/Pr ratio was highly and negatively correlated with CP (r = −0.77; *p* = 0.001), NDF (r = −0.75; *p* = 0.002), DM (r = −0.74; *p* = 0.002), OM (r = −0.73; *p* = 0.002), HC (r = −0.73; *p* = 0.002) and NFC (r = −0.71; *p* = 0.003), but was moderately and negatively correlated with ADF (r = −0.64; *p* = 0.010), NFC/NDF (r = −0.59; *p* = 0.019) and EE (r = −0.58; *p* = 0.024). In contrast, the GP was moderately and negatively correlated with HC (r = −0.58; *p* = 0.025), DM (r = −0.55; *p* = 0.033), OM (r = −0.55; *p* = 0.035), NFC (r = −0.54; *p* = 0.036), NDF (r = −0.54; *p* = 0.040) and CP (r = −0.53; *p* = 0.041).

The variables were clustered in the left and superior edges of the heatmap (Figure 1). The cluster analysis shows that variables are classified into two clusters, “A” and “B”. The cluster “A” included IVDMD and most of the ruminal fermentation parameters (GP, pH, Ac/Pr ratio, GY, acetate, CH_4_ production, butyrate, (Ac + Bu)/Pr ratio, and CH_4_ yield). On the contrary, the cluster “B” grouped the substrates’ chemical components (DM, OM, NDF, NFC, CP, HC, ADF, EE, NFC/NDF and TT) and the rest of the ruminal fermentation parameters (PF, NH_3_-N, total VFA and propionate). The analysis clearly separates the TT from IVDMD, and gas and CH_4_ outputs, but it does not separate the TT from NH_3_-N and total VFA production.

### 3.5. Principal Component Analysis

**Figure 2:**
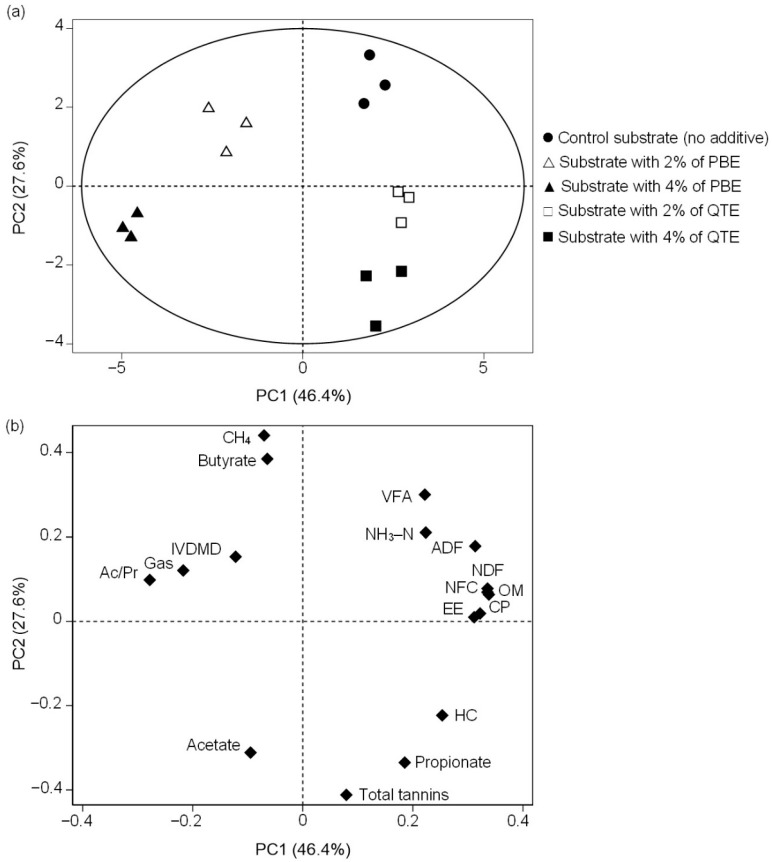
Principal component analysis of substrate composition and ruminal fermentation parameters after 24 h in vitro ruminal incubation. (**a**) Score plot and (**b**) loading plot projected on the basis of the two principal components (PC1 and PC2). For abbreviations, see Figure 1.

As a result of the PCA (Figure 2), 74.1% of the total variation was explained by the first two principal components. The first principal component (PC1; x-axis) with an eigenvalue of 7.90 accounted for 46.4% of the variance, while the second principal component (PC2; y-axis) with an eigenvalue of 4.70 explained 27.6% of the variance. The score plot (Figure 2a), used in the classification of the substrate, showed that PC1 clearly separated 2 major groups based on the plant extract; substrates with PBE were mostly in the negative range, whereas those from the CTL and substrates with QTE were in the positive range. Additionally, PC2 largely discriminated based on the concentration of the extracts; CTL was positioned in the positive range, whereas substrates supplemented with PBE and QTE at 4% DM were positioned in the negative range, distant from CTL. Nevertheless, substrates supplemented with PBE and QTE at 2% DM were mostly found in intermediate positions.

The loading plot (Figure 2b), which describes the relationship between variables, showed that the CP, EE, NDF, NFC and OM were clustered and positively correlated with PC1 and were loaded between ADF and the HC. On the other hand, CH_4_ production and butyrate proportion were positively correlated with PC2, whereas TT and acetate proportion were negatively correlated with this PC. In addition, the CH_4_ production had an opposite direction to TT.

## 4. Discussion

This study aimed to compare the PBE with a QTE, expecting to provide a comparable evaluation of the relative efficacy of the less-studied PBE on diet digestibility and the production of CH_4_, NH_3_-N and total VFA.

Although both PBE and QTE are polyphenolic extracts, PBE addition changed substrate composition, as shown by the PCA, since the only source of variation among the incubated substrates was the type and concentration of the plant extract. The inclusion of PBE decreased the DM of the substrate given its aqueous state (38.0% DM), and slightly increased the NDF of the substrate (from 30.0 to 31.3% DM with 4% PBE) even though the amount of mixed hay was decreased (from 600 to 576 g/kg DM). This probably occurred as a response to NDF concentration in the PBE (48.6% NDF), since the amount of mixed hay also decreased in the substrates supplemented with QTE; however, its NDF did not increase. As such, the increase in HC (from 8.00 to 9.70%) could be related to the increase in NDF and to a slight reduction in ADF.

The in vitro DM disappearance was reduced with QTE inclusion, although QTE had a greater relative degradation rate than PBE, suggesting that PBE only delays the initial fermentative activity. These results concur with studies in which phenolic compounds from oak (*Quercus robur* with *Q. petraea*) modulate rumen fermentation without causing any negative effects on DM digestibility [32] and with studies in which increasing concentrations of QTE decreased DM digestibility [33]. The use of forages with high tannin concentrations have been reported to reduce IVDMD [7] due to the capability of tannins to combine with minerals, proteins, cellulose, HC and pectins. Condensed tannins can also bind and inhibit different bacterial populations [2], cellulolytic microorganisms or their enzymes [15,16].

Both extracts decreased the rate of GP (*c*), although the lag phase was only decreased by QTE. In vitro GP and its kinetics depend on substrate chemical composition [34] and on ruminal microorganisms’ activity [3]. Rira et al. [6] and Brutti et al. [7] reported decreased in vitro GP using tannin-rich plant extracts as a consequence of tannins’ union to microorganisms and/or feed particles, thus inhibiting ruminal degradation. However, some tannin-tolerant microorganisms such as *Streptococcus gallolyticus*, a strain of *Prevotella ruminicola*, and a Gram-negative rod which belongs to the class *Proteobacteria* [35], could benefit from dietary tannins, thus increasing GP and decreasing the time to reach the asymptotic mean [14].

Methane per mL gas and per g incubated DM decreased with both extracts; however, asymptotic CH_4_ production only decreased with QTE, concurring with the decrease in the IVDMD. In addition, a negative correlation between CH_4_ production and TT was found, given that they were loaded on opposite sides of the PCA. Therefore, the reduced CH_4_ production could be related to the presence of polyphenols (tannins) in both plant extracts, confirming the effect of plant secondary metabolites on CH_4_ production, as reported in studies with valonea (*Q. aegilops*) [36], chestnut (*Castanea* sp.), sumach (*Rhus typhina*), mimosa (*Mimosa tenuiflora*), quebracho [4], tropical tree leaves [37,38], rambutan peel (*Nephelium lappaceum*) [39], red or white grape marc (*Vitis vinifera*) [40], Brazilian spinach (*Alternanthera sissoo*) [41] and agati (*Sesbania grandiflora*) [42]. These effects have been associated to the direct inhibition of methanogenic archaea [43]. Flavonoids are benzo–L–pyrone derivatives that have antimicrobial properties [41] by acting on the cell membrane and enzymatic activity [38]. However, tannin effects on CH_4_ can also be indirect, through the limitation of H supply for methanogens, by decreasing fiber digestibility [43] and consequently acetate and butyrate production, whose metabolic pathways release H [36], and by decreasing the protozoal populations [11], since protozoa provide H for methanogenesis [39]. The magnitude of tannins’ effects has also been related to their structure and molecular weight, which affect their ability to interact with fiber, proteins or microorganisms [6]. Since IVDMD was unaffected by PBE, it would be correct to suppose that the suppression of CH_4_ by PBE could be related to the decrease in ruminal microorganisms, such as archaea and protozoa, as opposed to QTE, which decreased the IVDMD. The lag phase was greater with PBE than QTE, although neither differed from CTL. Microbial colonization could be facilitated by substrates with a rapid degradation rate, which stimulate microorganism growth [44]. It is likely that PBE tannins bound nutrients in the substrate, delaying degradation and, therefore, CH_4_ production.

The production and composition of VFA depend on substrate quality, its availability, and rumen microorganisms [10]. At 24 h of incubation, total VFA production was decreased more by PBE than QTE, although QTE had a lower IVDMD. However, the IVDMD at 6 and 12 h of incubation was reduced by both extracts, suggesting a reduction in the production of VFA. Tannins can induce a reduction in VFA production by reducing rumen fiber digestion, as reported by Patra et al. [45] and Bueno et al. [2]. However, tannins have also been reported to reduce VFA production without affecting diet digestibility [46,47]. Our study did not determine the direct effects of both extracts on rumen microorganisms, but the differences between PBE and QTE can also be related to the capacity of microorganisms to adapt to the presence of polyphenols in the substrate, since the substrate components, additive concentrations and sampling times were identical. Generally, acetate and butyrate are produced by Gram-positive bacteria, while propionate is produced by Gram-negative bacteria [9], suggesting that PBE could inhibit Gram-negative bacteria [48]. In contrast, QTE supplementation tends to increase the propionate proportion, which is related to a reduced energy loss, given that propionate derived from the rumen is a glucose precursor [7] and the main source of metabolizable energy for ruminants [9]. In addition, changes in the VFA profile generally entail changes in CH_4_ production by alternative electron sinks [10], which could justify the decrease in CH_4_ production by QTE, since fermentation to propionate acts as a H sink [5], while acetate formation releases metabolic H [4]. Since acetate formation is related to fiber degradation [48], and both extracts increased acetate proportion without increasing the IVDMD, it is correct to suggest the presence of fermentable substances in the extracts, such as flavonoids, whose degradation contributes to acetate production [35]. The increase in the acetate proportion has also been reported in vitro by the addition of myrobalan (*Terminalia chebula*) tannin extract and grape seed [49], as well as in lambs supplemented with hop cones (*Humulus lupulus*), a plant rich in secondary compounds [50].

In this study, NH_3_-N production was decreased by at least 35.9% with PBE (2 and 4% DM) and 31.8% with QTE (4% DM), probably due to polyphenol concentration or types, specifically tannins. Tannins from sainfoin (*Onobrychis viciifolia*) [3,43], lingonberry (*Vaccinium vitis idaea*) [10,51], grape seed, sumach, valonea [49], oak bark (*Quercus cortex*) [51], quebracho [9], chestnut [52], birdsfoot trefoil (*Lotus corniculatus*) [53], pomegranate (*Punica granatum*) [54] or betel (*Piper betle*) [55] decrease NH_3_-N production by inhibition of protease activity [7], microbial deaminase and/or forming tannin-protein complexes at pH 6–7 in the rumen [5]. Furthermore, the decrease in total VFA production can be partially explained by the reduction in NH_3_-N production, since a portion of the VFA can be formed from the deamination of amino acids derived from proteolysis [56]. The average pH values with PBE and QTE are favorable for cellulolytic microorganisms [3]. These results concur with those of Khiaosa–Ard et al. [43] and Hassanat and Benchaar [36], who reported no effects on ruminal pH by tannin supplementation.

Differences observed between PBE and QTE effects in rumen fermentation may be due to TT concentrations in extracts (43.5 vs. 79.6 g kg^−1^ DM, respectively) and to the presence of different flavonoids in the polyphenolic PBE, such as luteolin, pinocembrin, catechin, procyanidin, gallocatechin, quercetin and taxifolin [19], as compared to QTE, which is primarily a source of tannins [5,12,14]. However, the effect of tannin extraction and purification procedures may also influence their activity as ruminal fermentation modulators [12], as well as the interaction between tannins, saponins, essential oils, or other metabolites present in the substrates [6] which were not determined in our study.

## 5. Conclusions

The findings of this study indicate that in forage diets (forage-to-concentrate ratio of 60/40), supplementation with the polyphenolic radiata pine bark extract (PBE) shares many of the effects of quebracho tannin extract (QTE) on ruminal fermentation, although the magnitude of its effects depends on concentration. With the supplementation of 4% DM, both extracts reduce CH_4_, total VFA and NH_3_-N productions, butyrate proportions, and increase acetate after 24 h of incubation. However, QTE, unlike PBE, reduces IVDMD and increases propionate proportion. On the other hand, the supplementation with 2% DM of PBE, unlike QTE, decreases the NH_3_-N and total VFA production, whereas QTE decreases CH_4_ production. PBE has the potential to be used as an environmentally friendly additive in ruminant diets not only for its effects on CH_4_ and NH_3_-N production, without reducing IVDMD, but also because it represents an alternative use for a very abundant by-product from the forest industry that is usually burned. Further research is warranted to verify our results under in vivo conditions and to clarify the effects of PBE supplementation on animal production, sustainability and environmental impact.

## Figures and Tables

**Table 1:** Ingredients and composition of the experimental substrates.

Item	Treatments				
	CTL	PBE2	PBE4	QTE2	QTE4
*Ingredients (g/kg dry matter)*					
Mixed hay of ryegrass and clover	600	588	576	588	576
Corn grain	250	245	240	245	240
Soybean meal	150	147	144	147	144
Pine bark extract	–	20	40	–	–
Quebracho extract	–	–	–	20	40
*Chemical composition (% dry matter unless otherwise noted)*					
Dry matter (% fresh basis)	90.00	79.00	69.80	89.70	89.70
Organic matter	92.80	92.30	92.20	92.30	92.60
Crude protein	20.40	20.60	21.00	21.10	20.50
Ether extract	1.91	1.72	1.89	1.79	1.91
Non-fibrous carbohydrates	40.50	38.90	38.00	39.30	40.50
Neutral detergent fiber	30.00	31.10	31.30	30.10	29.70
NFC/NDF	1.35	1.25	1.21	1.31	1.36
Acid detergent fiber	22.00	21.40	21.60	20.80	20.40
Hemicellulose	8.00	9.70	9.70	9.30	9.30

CTL = control substrate (no additive); PBE2 = pine bark extract (2% DM); PBE4 = pine bark extract (4% DM); QTE2 = quebracho extract (2% DM); QTE4 = quebracho extract (4% DM). NFC/NDF = ratio of non-fibrous carbohydrates to neutral detergent fiber.

**Table 2:** Effect of pine bark (PBE) and quebracho (QTE) extracts at different concentrations on in vitro dry matter disappearance (IVDMD), gas, and methane (CH_4_) production kinetics.

Item	Treatments					SEM	*p*-Value	Contrasts		
	CTL	PBE2	PBE4	QTE2	QTE4			1	2	3
IVDMD										
*B* (%)	56.30 ^AB^	57.00 ^AB^	58.70 ^A^	54.30 ^AB^	50.30 ^B^	1.57	0.040	0.643	0.009	0.816
*C* (h^−1^)	1.47 ^AB^	1.32 ^BC^	1.20 ^C^	1.50 ^AB^	1.52 ^A^	0.05	0.004	0.497	<0.001	0.789
*A*	0.44 ^AB^	0.38 ^AB^	0.34 ^B^	0.46 ^A^	0.48 ^A^	0.02	0.013	0.665	<0.001	0.913
*T*_MDR_ (h)	8.39 ^A^	7.60 ^AB^	6.26 ^B^	8.17 ^A^	7.93 ^A^	0.42	0.017	0.130	0.040	0.146
MDR (% h^−1^)	1.48 ^AB^	3.24 ^A^	4.09 ^A^	1.01 ^B^	0.88 ^B^	0.66	0.014	0.559	<0.001	0.832
Gas production										
*b* (mL g^−1^ incubated DM)	125.00	127.90	129.10	122.60	120.10	4.51	0.512	0.883	0.068	0.822
*c* (h^−1^)	0.19 ^A^	0.17 ^AB^	0.13 ^B^	0.17 ^AB^	0.16 ^B^	0.01	0.008	0.009	0.989	0.004
*Lag* (h)	1.78 ^AB^	1.79 ^AB^	2.45 ^A^	1.45 ^BC^	1.01 ^C^	0.16	0.008	0.326	0.024	0.589
*t*_1/2_ (h)	4.71 ^B^	4.96 ^B^	5.94 ^A^	4.65 ^B^	4.36 ^B^	0.13	<0.001	0.543	0.004	0.560
AR (mL g^−1^ incubated DM h^−1^)	11.40	10.80	10.50	11.30	11.00	0.31	0.331	0.183	0.125	0.300
CH_4_ production										
*b* (mg g^−1^ incubated DM)	9.37 ^A^	9.16 ^AB^	8.57 ^AB^	8.18 ^AB^	7.67 ^B^	0.42	0.039	0.073	0.019	0.079
*c* (h^−1^)	0.15	0.14	0.13	0.15	0.15	0.01	0.496	0.358	0.088	0.578
*Lag* (h)	2.37 ^AB^	2.54 ^AB^	3.21 ^A^	2.06 ^B^	1.75 ^B^	0.18	0.014	0.967	0.008	0.926
*t*_1/2_ (h)	6.08 ^AB^	6.20 ^AB^	7.06 ^A^	5.54 ^B^	5.25 ^B^	0.27	0.007	0.889	0.002	0.794
AR (mg g^−1^ incubated DM h^−1^)	0.74 ^A^	0.67 ^AB^	0.59 ^B^	0.72 ^A^	0.67 ^AB^	0.02	0.014	0.115	0.067	0.044

CTL = control substrate (no additive); PBE2 = pine bark extract (2% DM); PBE4 = pine bark extract (4% DM); QTE2 = quebracho extract (2% DM); QTE4 = quebracho extract (4% DM). *B* = potentially degradable fraction; *C* = relative degradation rate; *A* = constant factor of microbial efficiency; *T*_MDR_ = time of maximum degradation rate; MDR = maximum degradation rate; *b* = asymptotic gas or CH_4_ production; *c* = rate of gas or CH_4_ production; *Lag* = initial delay before gas or CH_4_ production begins; *t*_1/2_ = half–life; AR = average gas or CH_4_ production rate; SEM = standard error of the mean. ^A^^−^^C^ Different letters in the same row indicate significant differences (*p* < 0.05). The *p*-values are presented for the overall treatment effects and for orthogonal contrasts: 1 (CTL vs. plant extracts); 2 (PBE vs. QTE); and 3 (concentration of plant extracts at 0% DM vs. 2% DM vs. 4% DM).

**Table 3:** Effect of pine bark (PBE) and quebracho (QTE) extracts at different concentrations on in vitro dry matter disappearance (IVDMD), gas, and methane (CH_4_) outputs after 6, 12 and 24 h in vitro ruminal incubation.

Item	Treatments					SEM	*p*-Value	Contrasts		
	CTL	PBE2	PBE4	QTE2	QTE4			1	2	3
6 h										
IVDMD (%)	42.50 ^A^	39.60 ^AB^	38.00 ^B^	41.20 ^AB^	39.30 ^AB^	0.92	0.023	0.013	0.091	0.043
Gas (mL)	18.00 ^A^	14.90 ^B^	11.10 ^B^	18.00 ^A^	17.90 ^A^	0.84	<0.001	0.439	<0.001	0.065
g^−1^ incubated DM	38.60 ^A^	34.70 ^AB^	31.40 ^B^	38.00 ^A^	37.20 ^AB^	1.37	0.011	0.196	0.005	0.091
g^−1^ degraded DM	99.50 ^A^	91.00 ^AB^	80.20 ^B^	94.40 ^AB^	95.50 ^AB^	4.65	0.027	0.276	0.026	0.114
CH_4_ (mg)	1.05 ^A^	0.82 ^B^	0.59 ^B^	1.04 ^A^	1.03 ^A^	0.05	<0.001	0.254	<0.001	0.038
mL^−1^ gas	0.059 ^A^	0.055 ^AB^	0.052 ^B^	0.059 ^A^	0.057 ^AB^	0.001	0.013	0.218	0.004	0.087
g^−1^ incubated DM	2.31 ^A^	2.00 ^AB^	1.63 ^B^	2.20 ^A^	2.14 ^AB^	0.15	0.019	0.189	0.013	0.089
g^−1^ degraded DM	5.52 ^A^	5.00 ^AB^	4.07 ^B^	5.92 ^A^	6.02 ^A^	0.35	0.023	0.537	0.011	0.232
12 h										
IVDMD (%)	51.40 ^A^	46.80 ^AB^	45.10 ^B^	48.40 ^AB^	46.50 ^B^	1.30	0.004	0.001	0.144	0.083
Gas (mL)	38.50 ^A^	32.20 ^B^	29.60 ^B^	34.60 ^AB^	32.90 ^B^	1.26	0.003	0.003	0.035	0.100
g^−1^ incubated DM	82.50 ^A^	77.90 ^AB^	71.20 ^B^	76.50 ^AB^	70.80 ^B^	2.21	0.002	<0.001	0.574	0.003
g^−1^ degraded DM	160.70	159.70	153.80	147.20	148.40	7.20	0.279	0.060	0.109	0.744
CH_4_ (mg)	2.45 ^A^	1.95 ^B^	1.54 ^C^	2.24 ^AB^	2.13 ^AB^	0.07	<0.001	0.002	<0.001	0.006
mL^−1^ gas	0.064 ^A^	0.060 ^AB^	0.053 ^B^	0.065 ^A^	0.065 ^A^	0.002	0.001	0.788	0.001	0.073
g^−1^ incubated DM	5.25 ^A^	4.70 ^AB^	4.18 ^B^	4.78 ^AB^	4.59 ^AB^	0.16	0.007	0.004	0.157	0.047
g^−1^ degraded DM	10.23	9.65	9.32	9.89	9.84	0.42	0.655	0.404	0.377	0.643
24 h										
IVDMD (%)	60.10 ^A^	57.60 ^AB^	56.10 ^AB^	59.50 ^AB^	53.40 ^B^	1.49	0.041	0.131	0.809	0.021
Gas (mL)	56.20	49.20	46.20	54.00	51.20	3.26	0.130	0.203	0.154	0.303
g^−1^ incubated DM	120.70	116.20	117.20	114.00	112.10	5.83	0.743	0.219	0.456	0.475
g^−1^ degraded DM	192.50	199.00	205.00	194.30	205.80	6.84	0.369	0.180	0.958	0.117
CH_4_ (mg)	4.35 ^A^	3.63 ^B^	3.04 ^C^	3.88 ^AB^	3.65 ^B^	0.13	<0.001	<0.001	0.006	0.005
mL^−1^ gas	0.077 ^A^	0.073 ^AB^	0.066 ^B^	0.072 ^AB^	0.070 ^AB^	0.002	0.038	0.044	0.616	0.011
g^−1^ incubated DM	9.28 ^A^	8.78 ^AB^	8.19 ^B^	8.26 ^B^	7.81 ^B^	0.26	0.005	0.004	0.072	0.002
g^−1^ degraded DM	15.40	15.20	13.80	14.10	14.60	0.65	0.199	0.095	0.927	0.199

CTL = control substrate (no additive); PBE2 = pine bark extract (2% DM); PBE4 = pine bark extract (4% DM); QTE2 = quebracho extract (2% DM); QTE4 = quebracho extract (4% DM). SEM = standard error of the mean. ^A−^^C^ Different letters in the same row indicate significant differences (*p* < 0.05). The *p*-values are presented for the overall treatment effects and for orthogonal contrasts: 1 (CTL vs. plant extracts); 2 (PBE vs. QTE); and 3 (concentration of plant extracts at 0% DM vs. 2% DM vs. 4% DM).

**Table 4:** Effect of pine bark (PBE) and quebracho (QTE) extracts at different concentrations on in vitro ruminal fermentation parameters after 24 h of incubation.

Item	Treatments					SEM	*p*-Value	Contrasts		
	CTL	PBE2	PBE4	QTE2	QTE4			1	2	3
pH	6.48 ^AB^	6.52 ^AB^	6.54 ^A^	6.47 ^AB^	6.44 ^B^	0.02	0.031	0.534	0.828	0.003
NH_3_-N (mg dL^−1^)	11.95 ^A^	7.65 ^B^	7.14 ^B^	10.12 ^AB^	8.15 ^B^	0.85	0.006	0.002	0.063	0.006
PF (mg degraded DM mL^−1^ gas)	5.08	5.04	4.89	5.27	4.88	0.10	0.095	0.641	0.374	0.057
Total volatile fatty acids (m*M*)	520.90 ^A^	431.30 ^BC^	383.30 ^C^	475.50 ^AB^	454.90 ^B^	15.78	<0.001	0.002	0.004	0.003
Acetate (%)	38.50 ^C^	40.90 ^BC^	46.20 ^A^	40.80 ^BC^	42.20 ^AB^	1.09	0.003	0.025	0.175	0.007
Propionate (%)	30.20 ^B^	31.50 ^AB^	31.80 ^AB^	37.20 ^AB^	38.80 ^A^	1.89	0.016	0.125	0.002	0.316
Butyrate (%)	38.60 ^A^	37.80 ^A^	25.50 ^B^	23.30 ^B^	20.50 ^B^	2.16	0.001	0.062	0.041	0.013
Ac/Pr	1.20 ^AB^	1.20 ^AB^	1.38 ^A^	1.10 ^B^	1.14 ^B^	0.05	0.017	0.967	0.009	0.309
(Ac + Bu)/Pr	2.71 ^A^	2.20 ^AB^	2.15 ^AB^	1.70 ^B^	1.69 ^B^	0.19	0.003	0.010	0.001	0.042

CTL = control substrate (no additive); PBE2 = pine bark extract (2% DM); PBE4 = pine bark extract (4% DM); QTE2 = quebracho extract (2% DM); QTE4 = quebracho extract (4% DM). NH_3_-N = ammonia nitrogen; PF = partitioning factor; Ac/Pr = acetate-to-propionate ratio; (Ac + Bu)/Pr = sum of the acetate-and-butyrate-to-propionate ratio; SEM = standard error of the mean. ^A−^^C^ Different letters in the same row indicate significant differences (*p* < 0.05). The *p*-values are presented for the overall treatment effects and for orthogonal contrasts: 1 (CTL vs. plant extracts); 2 (PBE vs. QTE); and 3 (concentration of plant extracts at 0% DM vs. 2% DM vs. 4% DM).

## Data Availability

The data for this study are available on request from the corresponding author.
